# Identifying non-traditional stakeholders with whom to engage, when mitigating antimicrobial resistance in foodborne pathogens (Canada)

**DOI:** 10.1186/s13104-018-3279-8

**Published:** 2018-03-12

**Authors:** Shannon E. Majowicz, E. Jane Parmley, Carolee Carson, Katarina Pintar

**Affiliations:** 1 School of Public Health and Health Systems, University of Waterloo, 200 University Avenue West, Waterloo, ON N2L 3G1 Canada; 2 Centre for Foodborne, Environmental and Zoonotic Infectious Diseases, Public Health Agency of Canada, 160 Research Lane, Guelph, ON Canada; 3 Canadian Wildlife Health Cooperative, Department of Pathobiology, University of Guelph, 50 Stone Road East, Guelph, ON Canada

**Keywords:** Antimicrobial resistance, Food safety, Participatory epidemiology, Public health policy

## Abstract

**Objective:**

Antimicrobial resistance (AMR) is a critical public health issue that involves interrelationships between people, animals, and the environment. Traditionally, interdisciplinary efforts to mitigate AMR in the food chain have involved public health, human and veterinary medicine, and agriculture stakeholders. Our objective was to identify a more diverse range of stakeholders, beyond those traditionally engaged in AMR mitigation efforts, via diagramming both proximal and distal factors impacting, or impacted by, use and resistance along the Canadian food chain.

**Results:**

We identified multiple stakeholders that are not traditionally engaged by public health when working to mitigate AMR in the food chain, including those working broadly in the area of food (e.g., nutrition, food security, international market economists) and health (e.g., health communication, program evaluation), as well as in domains as diverse as law, politics, demography, education, and social innovation. These findings can help researchers and policymakers who work on issues related to AMR in the food chain to move beyond engaging the ‘traditional’ agri-food stakeholders (e.g., veterinarians, farmers), to also engage those from the wider domains identified here, as potential stakeholders in their AMR mitigation efforts.

## Introduction

Antimicrobial resistance (AMR) is a critical public health issue causing increased morbidity and mortality worldwide [1, 2]. Antimicrobial use (AMU) in any sector, including in humans and animals, on crops, in cleaning products, or through environmental contamination during manufacturing, creates selection pressures that favour the survival of microorganisms less affected by, or resistant to, the antimicrobial’s effects [3, 4]. In pathogenic bacteria, AMR leads to infections that are difficult to treat. Alarmingly, such bacteria are exhibiting more serious resistance, including to multiple antimicrobials simultaneously [5], and to the most important antimicrobials for human medicine, including fluoroquinolones, 3rd and 4th generation cephalosporins, and other drugs of last resort (e.g., colistin; [6]).

In pathogenic bacteria transmitted via food, such as *Campylobacter* and *Salmonella*, AMR is a complex issue involving interrelationships between people, animals, and the environment, and AMU in food-producing animals (whether for preventive, therapeutic, or growth promotion purposes) is a recognized contributor to resistant human infections [7, 8]. Thus, public health efforts to track the link between on-farm AMU and the emergence of AMR have been implemented, allowing the resistance profiles of pathogenic bacteria from food animals on-farm and at slaughter to be compared to profiles from food products at retail and from subsequent human infection [9–12].

In addition to the direct relationship between AMU and AMR in animals and humans, it is important to assess the role of broader, systemic drivers. To-date, such assessments have evaluated the contribution of governance and corruption to AMR in a variety of pathogens including those transmitted by food [13]; explored the role of regulation related to AMR in the environment [14]; and considered the impact of AMR policy actions that have yet to adequately address the economic situations of farmers [15]. Exploring additional factors such as these enables other stakeholders—beyond those traditionally involved from the medical, public health, veterinary medicine and agri-food sectors—to be identified and engaged in creating sustainable actions to reduce AMU and AMR, and ultimately maintain the effectiveness of antimicrobials for human and veterinary medicine. Therefore, our objective was to diagram the range of potential proximal and distal factors impacting, or impacted by, AMU and AMR along the Canadian food chain, and to use this diagram to identify additional stakeholders who are not currently engaged in the effort to reduce AMR in foodborne pathogens in Canada, with whom researchers and policymakers working on AMR in foodborne pathogens can engage in future transdisciplinary activities.

## Main text

**Fig. 1:**
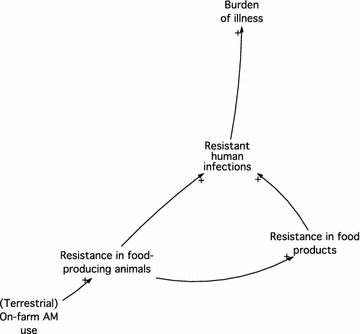
Model showing the traditionally-considered source of antimicrobial use and resistance in foodborne pathogens. *AM* antimicrobial; + represents the positive association between factors

We diagrammed factors related to AMU and AMR in the Canadian food chain, drawing on our own expertise in foodborne disease, AMR, and the Canadian food system, and informed by both group model building [16] and expert elicitation [17] approaches. We created a conceptual model that illustrated such factors, and the interrelationships between them where possible, through an iterative series of in-person brainstorming sessions. The model was created in Vensim^®^ PLE Plus for Macintosh (version 6.3; Ventana Systems, Inc.), and was drawn using a systems dynamics model format, which has been used for other public health issues to depict the underlying set of complex factors and interrelationships that drive the issue [18, 19]. We started by diagramming the most traditionally-considered source of AMR in the food chain, on-farm AMU in food-producing animals, and its links to AMR and human illness (Fig. 1). Next, we added other potential factors that could impact, or be impacted by, on-farm AMU. We continued expanding the conceptual model, by iteratively identifying additional factors, and adding specificity so that the factors aptly represented the Canadian food chain context, until no new changes were noted.

We then applied a case scenario to our draft model: the voluntary withdrawal of ceftiofur (a Category I antimicrobial, the most important category for human medicine [20]) in chicken hatcheries in the province of Québec, Canada, and the subsequent return to partial use, as described by Dutil et al. [10]. Briefly, 2003 data from the Canadian Integrated Program for Antimicrobial Resistance Surveillance showed that resistance to ceftiofur in human infections of *Salmonella* Heidelberg in the province of Québec was high, and tracked well against the level of resistance found in *Salmonella* Heidelberg isolated from retail chicken samples collected from Québec. Upon receiving these data in 2005, Québec hatcheries responded and voluntarily stopped using ceftiofur to treat and prevent *E. coli* omphalitis in chicks, and in 2006 levels of resistance in both *S.* Heidelberg infections in humans and *S.* Heidelberg isolated from retail chicken decreased. However, in 2007, Québec hatcheries returned to partial use of ceftiofur to treat and prevent *E. coli* omphalitis, and resistance in human infections and retail chicken began to increase. By 2013, levels of resistance had returned to the 2003 levels.

**Fig. 2:**
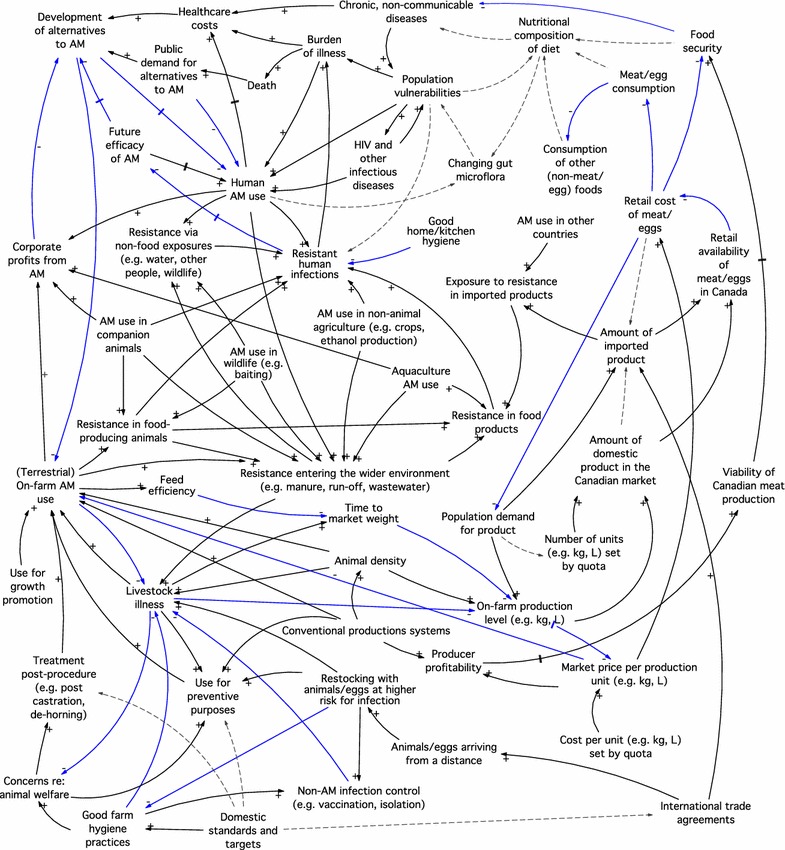
Final conceptual model, showing factors related to antimicrobial use and resistance in foodborne pathogens. *AM* antimicrobial; +/black arrow and −/blue arrow signs represent the positive and negative directions of association between factors (where possible); dashed lines show potential associations or complex pathways that cannot be summarized with a ± relationship; double hash marked lines show time-delayed pathways

Using this case scenario, we further refined our model by examining factors that may have: (1) been positively or negatively impacted when the voluntary withdrawal was in place; (2) been impacted had the withdrawal continued, either with or without any other changes in the underlying system of factors; or (3) contributed to the withdrawal being non-sustainable, that is, that might have driven hatcheries to start using ceftiofur again. For example, during the full withdrawal period we considered: did producers see a re-emergence of disease within flocks, and did this arise from the fact they were still producing chickens in the same manner as before, with no other changes in the broader production environment (e.g., economic drivers)? We used our knowledge of the industry and production practices to hypothesize about wider forces and added these factors to the model. We also examined the potential implications of the 2014 voluntary ban by the Canadian poultry industry, that “eliminates the preventative use of Category I antibiotics in Canadian chicken production” [21]. Through these explorations we added and removed factors and relationships, to yield the final conceptual model (Fig. 2).

We then used the model to identify potential stakeholders by determining the individuals and organizations who act upon the different factors in the model. We classified stakeholders by whether or not they had been commonly engaged by public health practitioners in mitigating and reducing AMU and AMR in the Canadian food chain (Table 1). Interestingly, this process allowed us to identify a wide range of potential stakeholders not typically engaged in the issue, many of whom exist as individual experts who may not necessarily be represented by organized groups. Historically, multidisciplinary approaches to mitigating and reducing AMU and AMR in the Canadian food chain context have engaged organizations; such entities are typically easier to identify and approach, and often represent large groups of individuals with a stake in the issue (e.g., Chicken Farmers of Canada, representing individual chicken farmers). Our model highlighted that such approaches to engagement, although important, may be missing key stakeholders for whom formal or organized entities do not exist, and that an important next step is to initiate conversations with a wider range of individuals with diverse expertise and experience, to explore potential roles and impacts of non-traditional stakeholders in the issue of AMR.

**Table 1:** Stakeholders in the issue of antimicrobial use and resistance in the Canadian food chain

Stakeholder	Traditionally engaged by public health organizations in the issue of AMR in the food chain?	
	Yes	No
Organizations	Federal Agriculture, Food, Health, and Trade Ministries Provincial Ministries of Agriculture, Health/Public Health Animal and Human Health Organizations Animal Industry Organizations Vet/Human Medical Associations Codex Alimentarius Commission Food and Agriculture Organization of the United Nations (FAO) World Organisation for Animal Health (OIE) World Health Organization (WHO) Drug Manufacturers (R&D, marketing)	Consumers, consumer advocacy groups Political organizations Urban, municipal planners
Individuals	Farmers, veterinarians	Nutrition, food security experts Farm, international market economists The public Educators, parents Health communication, program evaluation experts Lawyers Animal, human welfare advocates Academia, network/systems experts Political strategists, lobbyists Demographers Futurists, social innovators Environmentalists, urban agriculture workers

## Conclusions

Generating a conceptual model of the factors underlying AMR in foodborne pathogens was a useful process that allowed us to identify stakeholders who are not traditionally engaged by public health, to mitigate AMR in the food system. In addition to identifying stakeholders beyond the traditional agri-food partners, our model also identified that traditional methods for stakeholder engagement, that focus on engaging organizations, may be missing key stakeholders for whom formal or organized entities do not exist. Future efforts to engage a broader range of stakeholders are needed, in ways that allow for dialogue and exploration of how they act within and impact the system, in order to identify multi-pronged and sustainable approaches to mitigate and reduce AMR and its impacts across humans, animals, and the environment. Specifically, such dialogue is necessary to: (1) identify additional relevant model factors from domains like land use management, the media, access to health care and services; (2) explore other case scenarios to further identify factors that hold the current system ‘in place’ (e.g., support keeping the current level of AMU); (3) co-define potential courses of action, including roles and key influencers; and (4) explore potential ramifications, both intended and unintended, of possible actions. The ideal expansion of this work would involve revising the model to include all additional relevant factors and inter-relationships, and assigning quantities and functions to all factors and relationships, respectively, so that predictive modelling can occur. To achieve this, expert engagement with the range of stakeholders identified here is needed, to determine where quantitative data exist, and where approaches like expert elicitation are needed to specify such a model in the absence of empirical evidence.

## Limitations

We recognize that this work is subject to several limitations, most notably that it relies on our own expertise. However, this should not diminish the utility of the findings as a first depiction of the diversity of factors related to AMR in the Canadian food chain context, that allows us to identify additional domain experts with whom to engage in future model expansion and validation. Our approach was guided by expert elicitation, a method particularly useful for addressing questions that are difficult to answer via any other means [17, 22–25], that has been used previously to both qualitatively to rank pathways and build models [26, 27], and to produce quantitative estimates [24, 25, 28]. In the field of enteric pathogen source attribution, for example, when quantitative data are incomplete or unavailable, expert elicitation represents the only possible method for synthesizing knowledge about pathogen transmission [29]. In expanding the model presented here, expert elicitation involving diverse domain experts will undoubtedly be key to specifying the larger model structure (as we have begun, here), to identifying empirical evidence and data that can be used to quantify model components (e.g., to build structural equation models), and to determining values for model components where quantitative data are missing or incomplete.

We also recognize that taking a higher level systems view of AMR, as we have done here, necessitates ignoring nuance and detail, and reduces entire disciplines to a summary arrow or label in our model. This is another reason that domain experts across the factors identified here must now be engaged to ensure model completeness and accuracy, particularly given that we recognize our perspective is biased towards public health, foodborne disease, and veterinary medicine and livestock-associated factors. As well, to manage the size of this model, we focused on AMR in foodborne pathogens; however, micro-organisms transmitted via different pathways interact to shape the resistance landscape, and it will be important to explore how AMR in non-foodborne pathogens (e.g., AMR in *Neisseria gonorrhea*; [30]) may link or add to the factors identified here. Nevertheless, we feel that presenting our process, our model and our identified list of stakeholders provides a transparent starting point for more in-depth model building and stakeholder engagement processes, and that taking a broad approach to identifying model factors compliments existing models of AMR, that are often built specific to one domain and that typically focus on a limited number of proximate factors (e.g., [31]).

## Abbreviations

AMU: antimicrobial use
AMR: antimicrobial resistance
